# Scanning a microhabitat: plant-microbe interactions revealed by confocal laser microscopy

**DOI:** 10.3389/fmicb.2014.00094

**Published:** 2014-03-07

**Authors:** Massimiliano Cardinale

**Affiliations:** ^1^Institute of Plant Sciences, University of GrazGraz, Austria; ^2^Institute of Environmental Biotechnology, Graz University of TechnologyGraz, Austria

**Keywords:** plant-microbe interactions, cryptogams, confocal laser scanning microscopy (CLSM), endophyte, pathogen, GFP, DsRed, fluorescence *in situ* hybridization (FISH)

## Abstract

No plant or cryptogam exists in nature without microorganisms associated with its tissues. Plants as microbial hosts are puzzles of different microhabitats, each of them colonized by specifically adapted microbiomes. The interactions with such microorganisms have drastic effects on the host fitness. Since the last 20 years, the combination of microscopic tools and molecular approaches contributed to new insights into microbe-host interactions. Particularly, confocal laser scanning microscopy (CLSM) facilitated the exploration of microbial habitats and allowed the observation of host-associated microorganisms *in situ* with an unprecedented accuracy. Here I present an overview of the progresses made in the study of the interactions between microorganisms and plants or plant-like organisms, focusing on the role of CLSM for the understanding of their significance. I critically discuss risks of misinterpretation when procedures of CLSM are not properly optimized. I also review approaches for quantitative and statistical analyses of CLSM images, the combination with other molecular and microscopic methods, and suggest the re-evaluation of natural autofluorescence. In this review, technical aspects were coupled with scientific outcomes, to facilitate the readers in identifying possible CLSM applications in their research or to expand their existing potential. The scope of this review is to highlight the importance of confocal microscopy in the study of plant-microbe interactions and also to be an inspiration for integrating microscopy with molecular techniques in future researches of microbial ecology.

## Introduction

Plant-microbe interaction studies, including plant colonization by microbes, have benefitted from the development of high-throughput molecular methods, such as metagenomics and metatranscriptomics (Kint et al., 2010; Röling et al., 2010; Zhang et al., 2010; Jansson et al., 2012). Consequently, studies of microbe-host associations have become a core theme in microbial ecology, as their role for the macroscopic hosts was increasingly recognized. Omics methodologies based on the extraction of molecules (such as nucleic acid or proteins) directly from environmental samples, incremented tremendously the detection limit, thus broadening the spectrum of potentially targeted organisms to include also the rare microbiome. On the other hand, such methods have the disadvantage to lose the spatial information, since microbial cells are physically removed from their original location. For these reasons, methods allowing localization and visualization of microbes in microbe-host systems have also progressed during the past two decades, parallel to molecular microbiology methods. One of the frequently used approaches includes confocal laser scanning microscopy (CLSM) (Pawley, 2006). Plants, plant-like organisms, or fungi, are structurally complex and intricately linked with their substrates. For analyses of their interactions with microbes, CLSM has come in the prime of life as one of the standard techniques used. In this review I highlight the progresses achieved in understanding microbial interactions with plants and plant-like organisms using CLSM and image analysis, focusing on fluorescence *in situ* hybridization (FISH) and labeling with fluorescent proteins as common methods to specifically detect target organisms. As a direct method to study microorganisms, microscopy avoids the PCR biases typical of molecular methods, thus is best suited to accurately quantitate environmental microbes when a statistical approach is applied to image acquisition. I critically discuss this aspect together with the use of natural autofluorescence. Confocal image series contain an exceptional amount of potential information, but suitable methods for image analysis are required to exploit this potential. Here I show how different visualization methods can influence outcomes and conclusions of CLSM observations. Finally, I discuss future perspectives with CLSM and related techniques, and how their integration with molecular microbiology methods can contribute to a better understanding of host-microbe systems ecology. As already recognized explicitly for biofilms (Lourenço et al., 2012) I suggest the integration of CLSM with omics techniques as the optimal approach also in host-microbe interaction studies, both for laboratory-scale systems as well as for environmental samples.

## Basic principles: image acquisition, image analysis, and detection methods

CLSM is based on the detection of fluorescent light, but it differs from conventional epifluorescence microscopy by acquiring the fluorescent signal(s) exclusively from the focal plane as a pinhole excludes out-of-focus light. In addition, consecutive optical slices along the Z-axis of an image series (“confocal stack”) can be prepared for projections and three-dimensional reconstructions. Different signals can be acquired separately and then assigned to different colors for their discrimination in the images. Many CLSM instruments allow for addition of a (non-confocal) transmission light image to the confocal stacks. Confocal stacks can be analyzed in different ways, either by browsing the image series and selecting individual optical slices, or by sliding along any of the Euclidean axes to obtain X-, Y-, and Z-projections, respectively. Proprietary software tools can transform original fluorescent signals into artificial objects. Their surfaces are recognized by differences in fluorescence intensity (“isosurfaces”) and spheres. Such three-dimensional models facilitate precise localization of signals and intimate associations of organisms. For presentation, so-called time series can be compiled as short video clips, e.g., to move the viewing perspective, or to zoom regions of interest (flythrough).

Several freeware tools are available for qualitative and quantitative analysis of CLSM stacks. Although ImageJ was initially established for analysis of medical images (Schneider et al., 2012; http://rsbweb.nih.gov/ij/), several plugins were since then developed and applied for CLSM analyses of microbial communities. In plants these helped to analyze rhizosphere and phyllosphere communities (Iverson and Maier, 2009; Downie et al., 2012; Lee et al., 2012). Image surfer was developed with the specific purpose of imaging confocal stacks and it is not open to plugin implementation (Feng et al., 2007; http://imagesurfer.cs.unc.edu/). Nevertheless, it includes sophisticated visualization tools which allow the analysis of complex systems such as host-microbes interactions in the rhizosphere (Zachow et al., 2010). DAIME is a tool for quantitative analysis of complex microbial communities, such as biofilms, and also includes procedures for evaluation of fluorescence *in situ* hybridization probes (Daims et al., 2006; www.microbial-ecology.net/daime/).

CLSM allows the detection of three kinds of objects: (1) molecules, cells and tissues stained with one or more fluorochromes; (2) genetically modified organisms (GMO) that express fluorescent proteins; (3) autofluorescent cells, tissues and substrates. As autofluorescence of biological and synthetic substrates is usually considered as a negative aspect of CLSM images, efforts often aim toward avoiding autofluorescent signals (Lo Piccolo et al., 2010). As will be shown, autofluorescence may actually be a useful phenomenon for interpretation of the confocal images at least in the context of plant-microbes interactions.

FISH is most frequently used for visualization of microbial colonization patterns and community composition (Moter and Gobel, 2000; Amann et al., 2001). Owing to the direct visualization of target cells, FISH-CLSM can provide useful estimates of bacterial numbers in certain habitats, also because it avoids any quantification biases associated with methods based on cultivation or PCR (Bulgarelli et al., 2012). FISH is based on the hybridization of DNA-probes labeled with fluorochromes with the complementary target sequence. In most cases these are characteristic signature sequences of rRNA genes. Since specificity of the probes is defined by their sequence, it is ideally possible to detect a specific taxonomic range. Cautious interpretation of data is required with some probes which are known to have a lower specificity than ideally expected. Such information is included in databases for FISH probes, such as probeBase (Loy et al., 2003, 2007; http://131.130.66.201/probebase/).

Detection of mRNA targets is interesting for addressing functional questions, such as to understand the molecular bases of the mechanism(s) of interactions between beneficial microbes or pathogens and their respective host. However, low numbers of targets may impair detection with fluorescent FISH probes. Eventually, the signal can be increased by double labeling of oligonucleotide probes (DOPE-FISH; Stoecker et al., 2010) and by enzymatic amplification of the signal production, or by amplification of the target via *in situ* PCR (Ruppel et al., 2006).

FISH usually requires a preliminary fixation. Hence, the confocal images represent snapshots of the dynamic biological system, taken at the time of fixation. As fixation kills all cells, FISH staining generally does not allow any live imaging of cells, and separate samples fixed at different biological stages do not represent “true” time-lapse experiment. For live imaging (4D microscopy), fluorescent proteins produced in host- associated microorganisms after genetic transformation offer an alternative detection possibility. With this approach, time-lapse experiments can track the effects of substrates, growth enhancers and inhibitors. Genes coding for fluorescent proteins are usually inserted in plasmids successively cloned into competent cells but they can be also integrated chromosomally (Morschhäuser et al., 1998). Such proteins include green fluorescent protein (GFP), yellow fluorescent protein (YFP), and DsRed protein (Leveau and Lindow, 2002; Larrainzar et al., 2005). The use of the plasmids allows the insertion of additional genes, such as antibiotic resistance genes, useful to maintain the strain under selective growth. In fact, one of the biggest disadvantages of using the fluorescent proteins is their instability. Moreover, only the tagged strain can be visualized, which explains why GFP-tagged strains are usually applied in gnotobiotic systems, or used in microcosms with only one or few different microorganisms. Other plasmid constructs can include promoters upstream of the *gfp* gene, allowing the investigation of gene regulation by external factors such as the presence/concentration of chemicals (Rothballer et al., 2005).

Recently a new protocol for FISH without prior sample fixation was presented (Yilmaz et al., 2010). This method offers new and exciting perspectives for enabling simultaneous detection of FISH-stained natural populations and fluorescent protein-tagged strains. FISH was often coupled with other staining techniques. Many protocols have been developed; among others, Raman-FISH (Read et al., 2009), catalyzed reporter deposition-FISH (CARD-FISH; Pernthaler et al., 2002) and enhanced element labeling-FISH (EL-FISH; Behrens et al., 2008) address one of the most critical points of microbial ecology: to link identity and function of members of the natural microbial communities. These hybrid methods have not been used yet to study plant-microbes interactions.

## CLSM applied to host-microbe interactions

### Plants

CLSM in microbial ecology was first used by Schloter et al. (1993) to show the interactions between wheat roots and *Azospirillum brasilense* SP7, a plant growth promoting rhizobacterium (PGPR). In this case, the bacteria were stained with specific fluorescent labeled antibodies, and the authors pointed out the advantages of CLSM observations in comparison with those of traditional epifluorescence microscopy: they could precisely localize bacteria, root tissue and mucilaginous layer, and used XY or Z-scan images to show them. In this pioneering work, the authors could clearly show the great potential of CLSM in the field of microbial ecology. In the following 20 years, the number of scientific articles based on, or discussing, CLSM in plant-microbe interactions increased regularly, reached a plateau during the first decade of the new century of about 20 publications per year followed by a recent increase. This trend clearly reflects the technical development of new confocal systems. Applications of CLSM during these two decades ranged from studies of plant colonization to tracking the fate of inoculated strains. The studied hosts comprised vascular plants as well as cryptogams, such as mosses and lichens. A selection of relevant papers of the last 5 years is presented in Table 1 Supplementary material.

Plants provide a variety of microniches and surface types for bacterial colonization. Hence, the benefit of CLSM is to precisely localize the bacterial cells on plants. Bacteria were either detected on the rhizoplane, inside the root (endorhiza), in the apoplastic spaces, embedded in extracellular matrices, inside root cells, or inside the xylem vessels. Plant-associated bacteria not only use the microhabitats provided by the host as a house and eventually as substrate, but instead can actively shape them by modifying their development (Zamioudis et al., 2013).

Ahmed et al. (2010) described five distinct phases of root colonization by the Cyanobacterium *Leptolyngbya* within the same optical view (from root cell intrusion until total filling). As an alternative to multiple observations, this approach is only feasible when two prerequisites are met: (1) the target microorganism shows a stepwise colonization behavior with clearly discernible differences between the steps, and (2) its high density allows detecting different stages of colonization in close vicinity. CLSM offers the unique opportunity to elegantly show successive steps of microbial colonization as movies (Czymmek et al., 2007) or as image gallery (Prieto et al., 2011).

Zachow et al. (2010) studied interactions between fungal and bacterial beneficial strains in the root of sugar beet. The authors combined volume rendering and isosurface imaging to display the interactions between fungal hyphae and plant roots (Zachow et al., 2010), so providing an example of the CLSM versatility in imaging different organisms by mean of different visualization techniques. The results were interpreted in light of the microbial effects to the plant. It was concluded that neither endophytism nor direct contact with the pathogen was the discriminative feature of efficient biocontrol strains, so shedding light on their possible modes of action. Similarly, Maldonado-González et al. (2013) showed that, although not showing a direct contact with the pathogen, the biocontrol agent *Pseudomonas fluorescens* PICF7 was able to affect both the colonization patterns and the disease incidence of the tumor inducing *Pseudomonas savastanoi* NCPPB3335. Also in the phyllosphere of grapes, Gasser et al. (2012) showed that *Pantoea ananatis* BLBT1-08 efficiently controlled the plant pathogen *Botrytis cinerea*, although neither contact nor inhibition of conidia germination was observed.

Complementing CLSM with the identification of native beneficial bacteria in environmental samples sheds light onto the ecology of such strains in nature or under field conditions, as shown by Köberl et al. (2013) for *Bacillus* and *Streptomyces* in an arid ecosystem.

Fan et al. (2012) were able to show, in gnotobiotic systems, how the same rhizobacterium exhibited different colonization patterns on three different hosts, thus suggesting that every plant-microbe system is putatively unique and that it would be imprudent to draw general conclusions from results obtained with one system.

Bacterial-fungal interactions in the rhizosphere (such as mychorrizal systems) are ubiquitous and play an outstanding role for soil ecosystems, yet, they were not extensively studied by CLSM *in situ*. Mogge et al. (2000) studied the bacterial community on the ectomycorrhizal mantles of beech (*Fagus sylvatica*) and characterized its taxonomic composition by FISH. By using the fluorescence intensity as a quantitative reporter of metabolic activity, they demonstrated that incubation with nutrient sources such as yeast extract did not increase bacterial metabolism. In the rhizosphere of barley, intrahyphal occurrence of *Paenibacillus* and *Rhizobium* strains was proved with CLSM and correlated with their beneficial effect on the plant fitness (Sharma et al., 2008).

Rhizobia are unique among plant symbionts. They frequently induce development nodules as specific symbiont-hosting organs in certain plant lineages. Their infection process, elucidated at both phenotypic and molecular level, was also complemented by CLSM studies (Timmers et al., 1999; Haynes et al., 2004). *Burkholderia* strains (so called “β-rhizobia”) have been isolated from root nodules of several plants in the past. Such non-rhizobial symbionts were shown by CLSM to actually nodulate *Cyclopia* ssp. as well as the promiscuous legume *Macroptilium atropurpureum* (Elliott et al., 2007), and *Mimosa pigra* (Chen et al., 2005). CLSM revealed more bacterial species to be able to colonize the internal parts of root nodules, such as *Paenibacillus polymyxa* (Annapurna et al., 2013).

Kamilova et al. (2007) observed substantial differences in the interactions between a pathogenic fungus and its biocontrol agent *in vivo* and *in vitro*. While analysis *in vitro* does not suggest significant effects of *Collimonas fungivorans*, this bacterial strain exerts antagonistic activity *in vivo*. The authors illustrated their finding by CLSM and found no direct interaction between bacterial cells and fungal hyphae at microscopic scales. Olivain et al. (2006) followed colonization patterns of two different strains of the ascomycete *Fusarium oxysporum* (a pathogenic one and its antagonistic strain) in tomato roots. The image segmentation demonstrated that the two strains co-occur in the same areas of the root, which suggests competition for nutrients rather than a competition for space. This example shows how CLSM can contribute to the understanding of ecological relationships between microbes, including biological control. *In situ* auxine (indole-3-acetic acid) production of two *Azospirillum brasilense* strains was compared by using a fusion construct where the promoter of the gene *ipdC* (responsible for auxine synthesis) was integrated in a plasmid upstream of the *gfp* gene (Rothballer et al., 2005). Differences between the signal intensity of the two strains were then explained at molecular level by sequence analysis, which revealed the occurrence of a region exclusive for the most performing strain, probably involved in the regulation of expression. The same technique could be theoretically used for studying the *in situ* expression of any gene of interest.

Colonization of xylem vessels was shown, among others, for *Enterobacter gergoviae* (An et al., 2006), *Bacillus subtilis* (Ji et al., 2008), *Herbaspirillum frisingense* (Rothballer et al., 2008) and *Burkholderia terricola* (Gasser et al., 2011). Such observations are among the most challenging, because they are strongly dependent on the quality of the sectioning and the integrity of root anatomy. Clear identification of root tissues and preservation of the root anatomy provide the perfect background to investigate the localization of microorganisms in the rhizosphere or in the endorhiza. For example, cellulose autofluorescence revealed details of root anatomy, both with longitudinal (Maciá-Vicente et al., 2008) or transversal sections (An et al., 2006; Kutter et al., 2006).

Pathogenicity represents a special case within host-microbe interactions. The role of confocal microscopy can be relevant when complemented with molecular tools such as transformation and mutation. Mechanism(s) of interactions with the host can be understood and dynamics of infection processes elucidated. In the phyllosphere of lettuce the human pathogen *Salmonella enterica* intrudes the plant via the open stomata (Kroupitski et al., 2009). In this work, differential interference contrast images were overlapped with the confocal images, to visualize the inner tissues of the leaf, and co-occurrence of both bacterial signal (GFP) and chloroplasts (chlorophyll autofluorescence) revealed endophytically living bacteria in the plant's leaf tissue. Demonstrating the entry through the stomata is of critical importance, since this explains why conventional sanitation strategies based on soil treatment may fail to prevent pathogen infection of leafy vegetables. Li et al. (1999) demonstrated, by quantification of fluorescent signals derived by a promotorless GFP gene, that the expression level of *vir* genes in *Agrobacterium tumefaciens* varies during the infection process, also accompanied by changes in cell morphology. Newman et al. (2003) identified the vessel-to-vessel movement as the mechanism of infection responsible for the degenerative disease of *Vitis vinifera* induced by *Xylella fastidiosa* (an otherwise harmless endophyte).

Plant-microbes interactions in plant microbial fuel cells were studied by Timmers et al. (2012). In such devices, living plant roots provide electron donor for electricity generation in a mixed microbial community which generates electricity. The authors analyzed anode–rhizosphere bacterial communities of a *Glyceria maxima* (reed mannagrass) fuel cell. They found electrochemically active bacteria on the root surfaces, but at much lower abundance than on the graphite anode. As anaerobic cellulolytic bacteria neighbored the electrogenic bacteria, current production was enhanced by hydrolysis of cellulose.

### Cryptogams: mosses and lichens

In a study of bog mosses of the genus *Sphagnum*, Bragina et al. (2012) demonstrated by FISH-CLSM that the hyalocytes, i.e., dead moss cells which serve as water containers and are in direct contact with the external environment, are the preferred colonization sites. Further studies on the functions of such endophytes demonstrated their potential involvement on nitrogen fixation and methane degradation (Bragina et al., 2011, 2013). This suggests that these specific niches are not only water reservoirs. They might represent a sort of “micro-bioreactors” for nutrient production that supports the growth of the host, and may also exert direct ecosystem impact (Kip et al., 2010).

Lichens are traditionally considered as mutualistic symbioses of fungi and photoautotrophs (algae or cyanobacteria). Recent microscopic studies revealed high abundances of bacteria in these symbioses, comparable to those of rhizosphere soil and other microbial hot spots (Cardinale et al., 2008; Grube et al., 2009; Schneider et al., 2011). Counting of bacteria in confocal images of FISH-labeled bacteria helped to statistically evaluate the effect of environmental factors on the frequency of main bacterial phyla in different lichen species (Cardinale et al., 2012; this was one of the few cases in which data obtained with confocal microscopy were statistically assessed). Three-dimensional modeling of lichen microhabitats lead to reconsider the hypothesis of lichens as autonomous mini-ecosystems, this time including bacterial communities functionally adapted to the different thallus regions (Farrar, 1985; Grube et al., 2009; Cardinale et al., 2012).

## Critical issues in confocal microscopy of plant-microbe interactions

CLSM images are presented as either maximum projections or single optical slides. Therefore, it is necessary to know the thickness of the confocal stack as well as the Z-step dimension, in the case of maximum projections, for interpretating the images. In particular this is critical for studying endophytism, physical interactions and spatial arrangement of microbial populations. In the case of single optical slices, the thickness of sections should also be mentioned. If colonization is scant, low resolution may contribute to a misunderstanding of signals in the images unless critical interpretation confirms bacterial signal. Size and shape characters help to distinguish bacterial cells from autofluorescent objects in the same emission range. Conspecific microbial colonies are then recognized by discernible single cells with shared phenotype.

**Figure 1:**
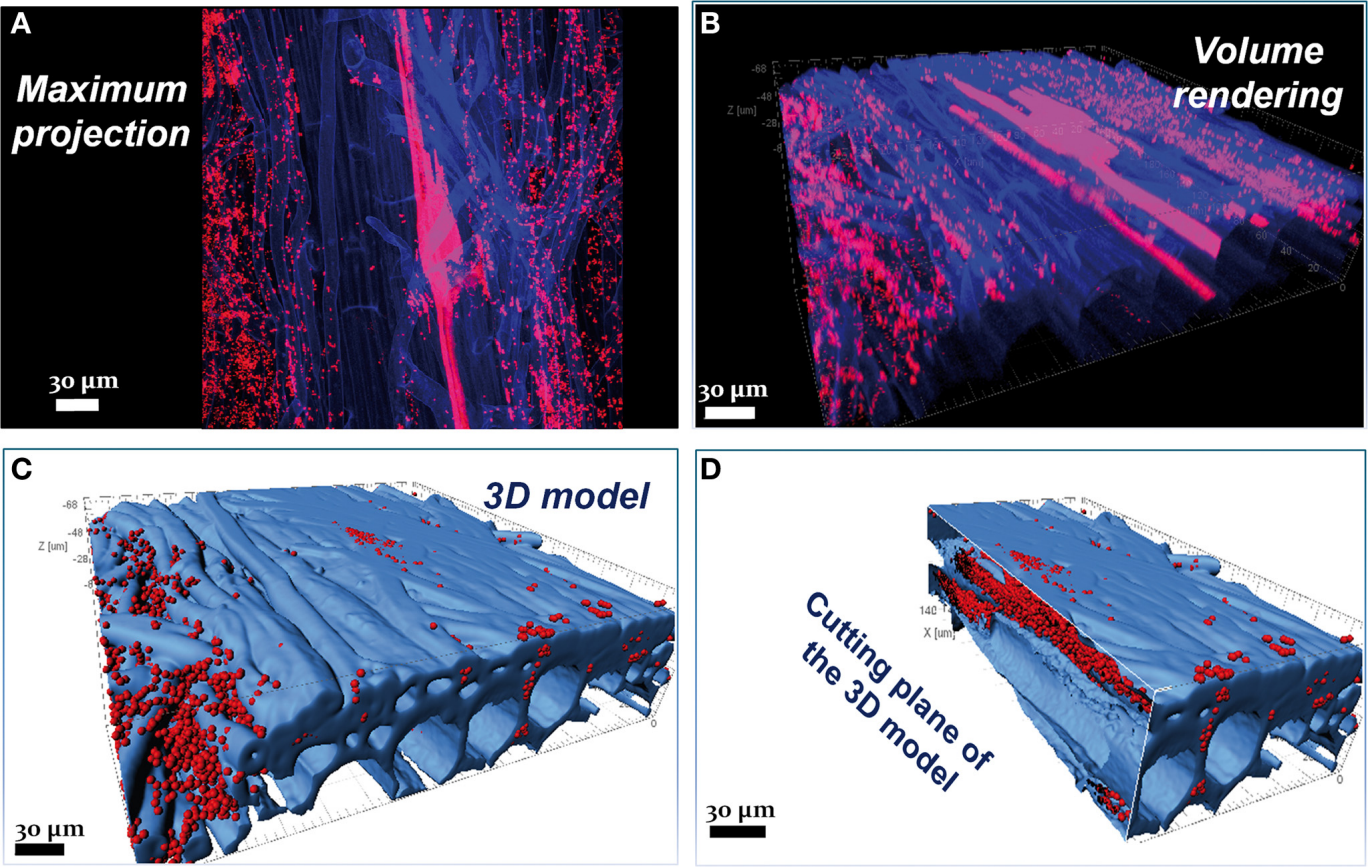
**Combination of FISH with autofluorescence**. Confocal images showing root colonization by the PGPR *Burkholderia terricola ZR2-12*. **(A)** In the maximum projection it is not possible to assess the colonization pattern of *Burkholderia terricola ZR2-12* (red) on this 3 weeks-old sugar beet root (blue); it is impossible as well to discriminate endophytism from ectophytism. **(B)** The volume rendering of the same confocal stack shows the cells colonizing the internal root tissues but only in the three-dimensional models **(C,D)** it appears clear that the same bacterium shows a double colonization style: ectophytic at the sides of the root **(C)** and endophytic, following the apoplastic spaces **(D)**; furthermore, the data from the three-dimensional models (number of spots, volume, etc.) can be easily retrieved and treated with statistics. This confocal stack has a thickness of 70.16 μm and was acquired with a Leica TCS SPE (Leica Microsystems GmbH, Mannheim, Germany) using the oil immersion objective Leica ACS APO 40.0x1.15. Z-step was 0.8 μm. Three-dimensional models were created with the software Imaris 7.3 (Bitplane, Zurich, Switzerland). Figure was prepared with Adobe Creative Suite version 3 (Adobe Systems Inc., San Jose, CA, USA).

Visualization tools are available for sophisticated analyses and improved interpretation of image data. As an example, the colonization pattern and the endophytism of the PGPR *Burkholderia terricola* ZR2-12 (Gasser et al., 2011) in the root system of sugar beet are impossible to assess in the maximum projection (Figure 1A), but become apparent only in the volume rendering (Figure 1B), three-dimensional modeling, and its cutting plane (Figures 1C,D, respectively). Such operations are possible with freeware Image Surfer (Feng et al., 2007) or professional software such as Imaris (Bitplane, Switzerland) and Amira (TGS Inc., US).

Autofluorescence is a typical phenomenon of CLSM with plant material. Pretreatments of the samples may help to reduce autofluorescence and prevent blurring of target signals in FISH experiments. In observations of plant-microbes interactions, however, the genuine autofluorescence can also help in precisely locating the microorganisms. Multichannel confocal systems with adjustment of detection ranges allow the dedication of one detection channel to the wavelength band of autofluorescence. This requires preliminary CLSM observations of unstained samples to find the lower and upper boundaries of autofluorescence absorption and emission (as well as its intensity). It is uncommon that a wide emission spectrum of the autofluorescence prevents application of suitable fluorochromes for staining of target microorganisms. It otherwise happens that autofluorescence is relatively weak. Signal accumulation during the image acquisition followed by a digital improvement by image post-processing can then help to suitably visualize an autofluorescent host matrix. Figure 1 gives an example of how the autofluorescence of sugar beet roots can be exploited to image the microhabitat of microbes as a reliable three-dimensional model.

**Figure 2:**
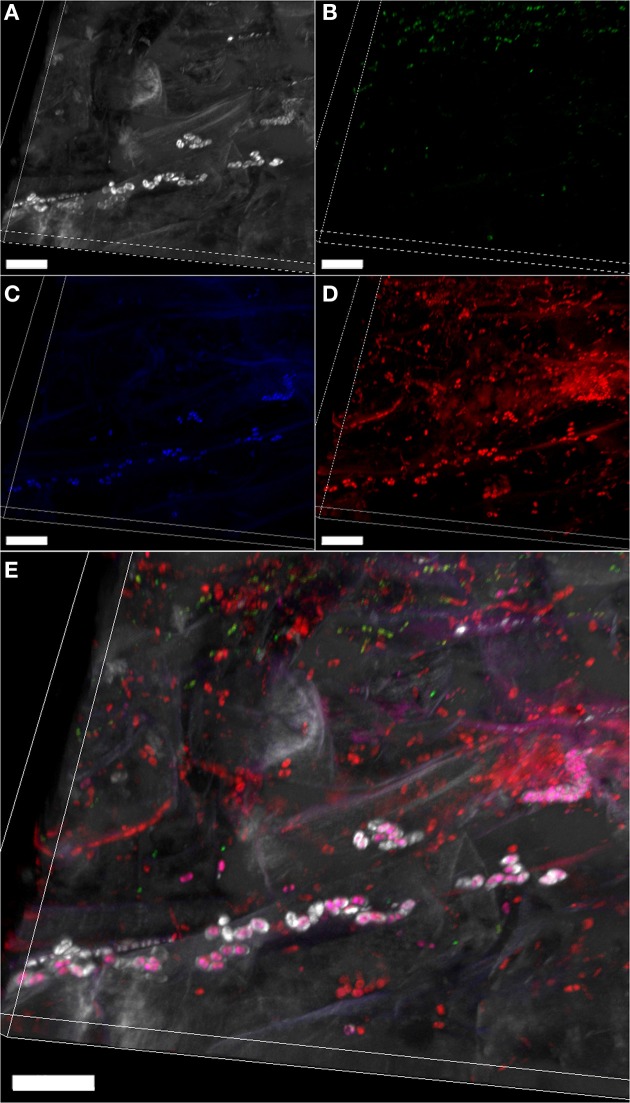
**Combination of FISH with histochemical staining**. Volume rendering of a confocal stack showing the bacterial colonization of salad root (*Lactuca sativa*) by the native bacterial community, stained by FISH. Gammaproteobacterial **(A)**, betaproteobacterial **(B)**, and other bacterial cells **(C)** (green, blue, and red, respectively) stained with the FISH probes Gam42a (Cy5-labeled), Bet42a (ATTO488-labeled) and EUBMIX (Cy3-labeled), respectively. **(D)** Compounds and tissues stained with calcofluor white (0.15% in H_2_O, 15 min incubation) appear gray. **(E)** Overlap of images **(A–D)**; yellow, Gammaproteobacteria; pink, Betaproteobacteria; red: other bacteria; gray: compounds surrounding some Betaproteobacteria stained by calcofluor white. Scale bars: 20 μm. Confocal stack has a thickness of 16.99 μm, and was acquired with a Leica TCS SPE (Leica Microsystems GmbH, Mannheim, Germany) using the oil immersion objective Leica ACS APO 40.0x1.15. The Z-step was 0.38 μm. Volume rendering was created with the software Imaris 7.3 (Bitplane, Switzerland). Figure was prepared with Adobe Creative Suite version 3 (Adobe Systems Inc., CA, USA).

Autofluorescence was already used for structural analysis of biofilm (Muñoz-Egea et al., 2013); however in case of weak autofluorescence of the host structures, histochemical staining can be coupled with FISH to enhance the signal of the host tissues supporting the microbial communities. A suitable staining is the Calcofluor white, which stains α 1-4-glucans characterizing many plant- and fungal cell walls as well as certain components of microbial outer layers (Figure 2). This hybrid approach allows distinguishing between structurally different populations within the same taxon, as shown for Betaproteobacteria in Figure 2.

## Quantitation of CLSM data

Microscopy is applied often for qualitative description of both complex populations and single species (for example pathogens or PGPR) and their localization. Indeed, there is only a handful of scientific papers on plant-microbes associations where CLSM data were analyzed quantitatively (e.g., Pivato et al., 2008; Iverson and Maier, 2009; Cardinale et al., 2012), even though the direct *in situ* observations could complement the PCR-based approach and even reveal PCR biases (Bulgarelli et al., 2012; Cardinale et al., unpublished data). However, certain factors can strongly limit the possibility of statistical approaches with CLSM images, even after it has been verified that detected signals represent target objects and not artifacts. For example, strain specific variation for species rich communities cannot be resolved by CLSM. It is thus advisable to complement CLSM data by other approaches, such as deep 16S rRNA gene amplicon sequencing, or metagenome sequencing, which both deliver suitable information for assessment of community structures as well as for evaluation of alfa- and beta-diversity.

**Figure 3:**
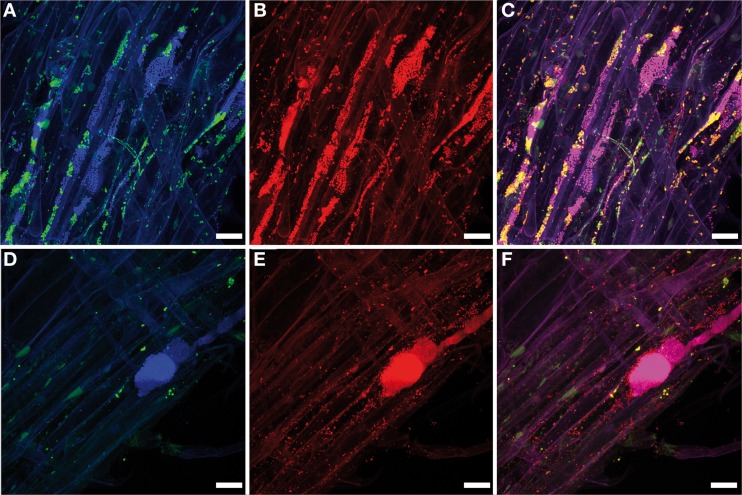
**Microbial interactions in the rhizosphere**. Maximum projections of a confocal stack showing the colonization pattern of salad root (*Lactuca sativa*) by the native bacterial community stained by FISH. **(A,D)** Gammaproteobacterial and betaproteobacterial cells (green and blue, respectively) stained with the FISH probes Gam42a (Cy5-labeled) and Bet42a (ATTO488-labeled), respectively. **(B,E)** All bacterial cells (red) stained with the FISH probe EUB338-MIX (Cy3-labeled). **(C,F)** Overlap of images **(A,B,D,E)**, respectively; yellow, Gammaproteobacteria; pink, Betaproteobacteria; red: other bacteria. Different taxa do not share the habitats, but instead colonize microniches of the rhizoplane dominantly, excluding each other (see text for more explanations). Scale bars: 20 μm. Confocal stacks **(A,C,D,E)** have a thickness of 30.72 and 37.26 μm, respectively, and were acquired with a Leica TCS SPE (Leica Microsystems GmbH, Mannheim, Germany) using the oil immersion objective Leica ACS APO 40.0x1.15. The Z-step was 0.5 μm. Maximum projections were created with the software Imaris 7.3 (Bitplane, Zurich, Switzerland). Figure was prepared with Adobe Creative Suite version 3 (Adobe Systems Inc., San Jose, CA, USA).

Microbial cells are unevenly distributed on their plant hosts. Therefore, the values of density of a certain host-associated microbial community does not inform about its actual dispersion across the host. Figure 3 shows an example of how the bacterial community associated with lettuce root can be differentially dispersed: in the first example (**A–C**) the community spreads evenly over the root surface, but different populations (Betaproteobacteria and Gammaproteobacteria) are unevenly distributed since it is possible to find regions exclusively colonized by one or the other group; the second example (**D–F**) shows a more drastic situation: only a big colony of Betaproteobacteria, surrounded by unidentified bacteria was detected, while the Gammaproteobacteria appear as single cells evenly distributed over the root; some regions of the root are almost bacteria-free. Such features can hardly be assessed statistically. In theory, a dispersion coefficient can be calculated for any host-associated microbial community provided that a sufficient number of confocal stacks (randomly acquired throughout the specimen) are analyzed (Ford and Harvey, 2007). The variance of different bacterial taxa *in situ* can be due to different growth strategies or even death rates. The host actively participates to trigger bacterial communities in the rhizosphere by root exudation (reviewed by Dennis et al., 2010) and a role in shaping its genetic structure was also suggested (Mølbak et al., 2007).

Bianciotto et al. (2004) demonstrated the vertical transmission of a bacterial endophyte of the arbuscular michorrizal fungus *Gigaspora margarita* through 4 generations of axenic culture. A statistical approach using CLSM allowed proving that the density of intrasporal bacteria strongly diminished from Generation 0 to Generation 4. This approach was based on manual counts of bacterial cells within 100 × 100 μm squares on single 3 μm-thick optical slices. The total number of detected cells for all the 7 optical slides of each confocal stack represented the density expressed as bacteria^*^mm^−3^. In different approaches the bacterial density is measured as colony forming units (CFUs, cultivation-dependent approaches) or as gene copy number (cultivation-independent approaches, q-PCR) per gram of host; thus a direct comparison with the CLSM results (volumetric values) is not possible. In order to directly compare CLSM data with data obtained by cultivation and q-PCR, it would be suitable to convert observed volumes into respective weights of sample. I and colleagues developed the “Delta-volume method” to express the density of bacteria detected by CLSM in lichen hosts as number of cells per gram of lichen thallus (Cardinale et al., 2012). To achive this, a subsample of the lichen specimens fixed for FISH-CLSM was immersed into a graduated tube partially filled with water: the difference in the volume was recorded (Delta-volume) and then the specimen was dried out and weighted. The ratio Delta-volume/weight was then used to convert the values expressed as bacteria^*^mm^−3^ (obtained by FISH-CLSM) into bacteria^*^g^−1^ lichen dw. This method might be applicable to every kind of environmental sample.

## Combination with other microscopic techniques: current state and perspectives

The combination of CLSM with other microscopic methods could offer additional advantages. In fact, the resolution of confocal microscopy, although higher than conventional light microscopy, is constrained by the optical limitations of the light microscopy; coupling fluorescent microscopy with cryo-electron microscopy in a correlative approach offers a possibility to first localize regions of interest or target objects and then visualize them at nanometric resolution (Sartori et al., 2007; Jahn et al., 2012). Coupling CLSM with a scanning probe system (such as an atomic force microscope—AFM) is another correlative approach which has been used for medical sciences but not yet for plant-microbes interactions (Haupt et al., 2006). Although an efficient CLSM-AFM protocol could be difficult to optimize (due to the fact that AFM works properly only with relatively flat specimens), it has anyway a potentiality to deliver deep structural information not available with CLSM only, such as interaction forces between beneficial microorganisms, pathogens and hosts. Correlative microscopy that combines FISH-CLSM with nanoSIMS could be particularly interesting, as this may ideally provide information on functional contributions of individual groups of bacteria. This has not yet been achieved and, until now, nanoSIMS has still rarely been used for studying plant-bacteria interactions. Clode et al. (2009) used this approach to visualize differential partitioning of ^15^NH^+^_4_ between plant roots and native soil microbial communities at the submicron scale.

## CLSM analysis as guidance for downstream experiments

**Figure 4:**
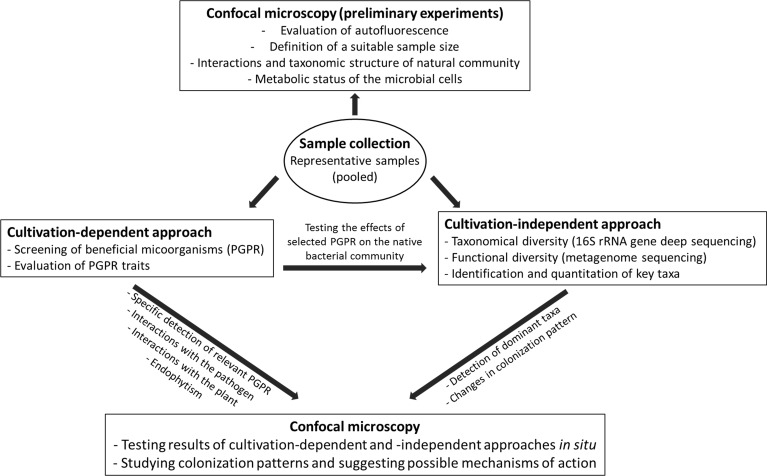
**Integration of CLSM with other techniques**. The workflow shows the combination of CLSM with other methods for plant-microbes interactions studies. A case study of PGPR is used in this example.

CLSM has been used to complement studies with other methods, such as deep sequencing or quantitative-PCR. Yet, the analysis of the native microbial communities using CLSM can help in hypothesis development and testing, and for proper sample size estimation for subsequent experiments. This includes the role of environmental factors on colonization patterns (Figure 4). Otherwise FISH-CLSM could guide the focus to particular bacterial groups in subsequent culture-dependent or culture-independent studies: in the case of lettuce root colonization (Figure 3), different taxa do not share the same site, but instead dominate in microniches of the rhizoplane and exclude each other (Figures 3A–C), and hence form locally extremely dense colonies (Figures 3D–F). The following hypotheses could result from these observations: (**A**) Lettuce roots either release specific exudates at microscale resolution, or concentrate them at particular sites on the root surface; (**B**) Different bacterial species arrive at different times, and the initial colonies exclude the following; (**C**) Finally, in case these bacteria are simultaneous colonizers, some species are locally enriched by faster growth. These hypotheses can be tested with specifically designed inoculation experiments under controlled conditions of growth.

## Conclusions and perspectives

Research of host-microbes systems requires a polyphasic approach to unravel the complexity of their interactions and ecological significance. Still, direct qualitative and quantitative information of bacterial colonization and its variation on the hosts' structures is only possible through direct visualization *in situ* and therefore CLSM serves as a central technology in such studies. The intrinsic variance of this information needs to be properly assessed by a statistical approach, to gain new and deeper insights into the stability and plasticity of host associated microbes in a changing environment.

Several other exciting microscopic techniques emerged over the past few years, e.g., Coherent Anti-Stokes Raman Scattering (CARS, Cheng et al., 2002), Multi-Isotope Imaging Mass Spectrometry (MIMS, McMahon et al., 2006), or Stimulated Emission Depletion Microscopy (STED, Westphal et al., 2008). However, these still depend on substantial infrastructure and their applicability to study a broader range of environmental samples has still to be shown. FISH-CLSM not only remains as a widely applicable methodology for studying plant-microbe interactions, but can be extended and complemented by other microscopic techniques.

In the last few years, microbial ecology was revolutionized by the advent of the deep-sequencing as a tool affordable for every laboratory. This was somehow similar to what happened in the 90 years, when fingerprinting techniques allowed for the first time the study of total microbial communities, including uncultivated organisms. Once more, the effect was that the scientists' attention was focused on the sequence-based information delivered by the new techniques and microscopy was overshadowed. Here I showed the critical role that microscopy (especially CLSM) had in the understanding of the processes. Localization at microscale, colonization pattern or cell-cell interaction, are not detectable by cultivation, fingerprinting, or deep-sequencing analysis, yet being the basic processes of plant-microbe interactions. High-resolution microscopy, coupled with suitable visualization and statistical methods, still represent the optimal tool that can provide such information and represents the best suited method to validate the results of molecular analysis in microbial ecology studies.

### Conflict of interest statement

The author declares that the research was conducted in the absence of any commercial or financial relationships that could be construed as a potential conflict of interest.
