# Supplementary material for: Scanning a microhabitat: plant-microbe interactions revealed by confocal laser microscopy
Source: Front Microbiol. 2014 Mar 7;5:94. doi: 10.3389/fmicb.2014.00094 (PMC3945399; doi:10.3389/fmicb.2014.00094)
Supplement: Supplementary file 1 [file DataSheet1.DOCX]

Table 1 (Supplementary material). Relevant works in the last 5 years in which confocal laser scanning microscopy was applied to plant-microbe interactions.

| Article | System studied | Staining method | Results and conclusions |
| --- | --- | --- | --- |
| Ahmed et al., 2010 | Rhizosphere - cyanobacteria | DTAF, autofluorescence | Endophytic behavior of some strains |
| Annapurna et al., 2013 | Soybean root nodules − *Paenibacillus polymyxa* | GFP - autofluorescence | Nodule colonization by a non-rhizobial bacterium |
| Bragina et al., 2011 | *Sphagnum* – native bacterial community | FISH, autofluorescence | Colonization pattern |
| Buddrus-Schiemann et al., 2010 | Rhizosphere – bacterial inoculant - indigenous bacterial community | GFP – FISH − autofluorescence | Effect of native bacteria on the inoculant colonization |
| Bulgarelli et al., 2012 | *Arabidopsis* rhizoplane | CARD-FISH | Incongruence with the deep-sequencing data: PCR biases revealed |
| Cardinale et al., 2008 | lichens – native bacterial community | FISH - autofluorescence | Formation of biofilm-like structures on fungal surfaces; colonization pattern |
| Cardinale et al., 2012 | lichens – native bacterial community | FISH - autofluorescence | Effect of different environmental/intrinsic factors on both the bacterial abundance and the community structure |
| Elliot et al., 2007 | Legume nodules – *Burkholderia* spp. | GFP - autofluorescence | Detection of bacteria inside the nodules |
| Fan et al., 2012 | Rhizosphere/root – *Bacillus amyloliquefaciens* | GFP - autofluorescence | Different colonization pattern of different plants |
| Fürnkranz et al., 2012 | Pumpkin anthosphere – bacterial inoculants | FISH - autofluorescence | Colonization pattern; presence of bacteria on pollen grains |
| Gasser et al., 2011 | Rhizosphere/root – *Burkholderia* inoculant | DsRed –autofluorescence | Dynamic of colonization; endophytic behavior; colonization of xylem vessels |
| Gasser et al., 2012 | Phyllosphere - *P. ananatis* - *Botrytis cinerea* | GFP - autofluorescence | Insights into the mechanisms of biocontrol |
| Grube et al., 2009 | lichens – native bacterial community | FISH - autofluorescence | Different colonization patter of lichen species; assessment of density and taxonomic structure |
| Iverson & Maier, 2009 | Rhizosphere – compost - indigenous bacterial community | FISH - autofluorescence | Positive effect of compost on root colonization |
| Ji et al., 2008 | Seedlings - *Bacillus subtilis* | GFP | Dynamic of colonization |
| Kaestli et al., 2011 | Grasses - *Burkholderia pseudomallei* | FISH - autofluorescence | Potential spread by grazing animals |
| Köberl et al., 2013 | Rhizosphere – *Bacillus*  and *Streptomyces* | FISH - autofluorescence | Rhizosphere competence of BCAs |
| Kroupitski et al., 2009 | Phyllosphere - *Salmonella enterica* | GFP - autofluorescence | Penetration of *Salmonella* trough the stomata |
| Liu et al., 2007 | Rhizosphere - *Comamonas* | GFP - propidium iodide | Colonization pattern; endophytic behavior |
| Long et al., 2008 | Rhizosphere – bacterial inocula | GFP | Intracellular colonization |
| Maciá-Vicente et al., 2008 | Rhizosphere/root - *Fusarium equiseti* -*Pochonia chlamydosporia* | GFP - autofluorescence | Root penetration by hyphae |
| Maldonado-González et al., 2013 | *Pseudomonas fluorescens - Pseudomonas savastanoi* - olive knots | GFP – RFP − Autofluorescence | Interaction BCA – pathogen - plant |
| Park & Kremer, 2010 | Root - *Pseudomonas fluorescens* | Immunofluorescence - autofluorescence | Influence of amendments on root colonization |
| Paungfoo-Lonhienne et al., 2010 | Root – *E. coli* -  *S. cerevisiae* | GFP | Bacterial “uptake” from plant root |
| Mercado-Blanco & Prieto | Olive Roots *- Pseudomonas* spp. | DsRed - GFP | Role of root hairs in the endophytic colonization of inner root tissues |
| Pivato et al., 2008 | Rhizosphere/root - *Pseudomonas fluorescens* - *Glomus mosseae* | Immunofluorescence | Different “organization types” between mychorrizal and non-mychorrizal roots |
| Pliego et al., 2008 | Rhizosphere/root – *Pseudomonas* (2 strains). - *Rosellinia necatrix* | GFP - autofluorescence | Different colonization pattern and different ability to adhere to fungal hyphae |
| Prieto et al., 2011 | Rhizosphere/root – *Pseudomonas* (2 strains) | EGFP – GFP - autofluorescence | Dynamic of colonization, endophytic behavior, simultaneous colonization |
| Rothballer et al., 2008 | Rhizosphere/root – *Herbaspirillum frisingense* | GFP - autofluorescence | Endophytic behavior, colonization of vessels |
| Ryan et al., 2009 | Plant root – *Stenotrophomonas* *rhizophila* | DsRed - autofluorescence | Endophytic behavior |
| Schmid et al., 2009 | Plant root – *Cronobacter sakazakii* | GFP – FISH - autofluorescence | Colonization pattern; endophytic behavior |
| Sharm et al., 2008 | *Piriformospora indica/Sebacina vermifera − Paenibacillus/Acinetobacter/Rhodococcus* | FISH - autofluorescence | Intrahyphal occurrence |
| Tharek et al., 2011 | Plant (root, stem, leaf) – bacterial inoculants (N_2_-fixing) | GFP –autofluorescence | Ascending endophytic migration |
| Verginer et al., 2010 | Phyllosphere – *Methylobacterium extorquens* | GFP –autofluorescence | Colonization pattern |
| Wu et al., 2008 | Rhizosphere - *Pseudomonas putida* | FISH –autofluorescence | Biofilm formation and gene expression *in situ* |
| Zachow et al., 2010 | Rhizosphere – bacterial and fungal inocula | GFP – DsRed - autofluorescence | Colonization pattern; endophytic behavior; selective germination of spores |

Additional bibliography for Table 1 (Supplementary material)

Buddrus-Schiemann, K., Schmid, M., Schreiner, K., Welzl, G., and Hartmann, A. (2010). Root colonization by *Pseudomonas* sp. DSMZ 13134 and impact on the indigenous rhizosphere bacterial community of barley. *Microb. Ecol.* 60, 381–393.

Fürnkranz, M., Lukesch, B., Müller, H., Huss, H., Grube, M, and Berg, G. (2012). Microbial diversity inside pumpkins, microhabitat-specific communities display a high antagonistic potential against phytopathogens. *Microb. Ecol.* 63, 418–428.

Kaestli, M., Schmid, M., Mayo, M., Rothballer, M., Harrington, G., Richardson, L., Hill, A., Hill, J., Tuanyok, A., Keim, P., Hartmann, A, and Currie, B. J. (2012). Out of the ground, aerial and exotic habitats of the melioidosis bacterium *Burkholderia pseudomallei* in grasses in Australia. *Environ. Microbiol.* 14, 2058–2070.

Liu, L., Jiang, C. Y., Liu, X. Y., Wu, J. F., Han, J. G., and Liu, S. J. (2007). Plant–microbe association for rhizoremediation of chloronitroaromatic pollutants with *Comamonas* sp. strain CNB-1. *Environ. Microbiol.* 9, 465–473.

Long, H. H., Schmidt, D. D, and Baldwin, I. T (2008). Native bacterial endophytes promote host growth in a species-specific manner; phytohormone manipulations do not result in common growth responses. *PlosOne* 3, e2702.

Lu, Y., Rosencrantz, D., Liesack, W., and Conrad, R. (2006). Structure and activity of bacterial community inhabiting rice roots and the rhizosphere. *Environ. Microbiol.* 8, 1351–1360.

Mercado-Blanco, J., and Prieto, P. (2013). Endophytic lifestyle of biocontrol strains of *Pseudomonas* spp. in olive roots, in Molecular microbial ecology of the rhizosphere, Volume 1 & 2 (ed F. J. de Bruijn), John Wiley & Sons, Inc., Hoboken, NJ, USA.

Park, K. C., and Kremer, R. J. (2010). Establishment of an inoculated bacterial strain on plant root surfaces in soils with different microbial community. *J. Korean Soc. Appl. Biol. Chem.* 53, 379–383.

Paungfoo-Lonhienne, C., Rentsch, D., Robatzek, S., Webb, R. I., Sagulenko, E., Näsholm, T., Schmidt, S., and Lonhienne, T. G. A. (2010). Turning the table, plants consume microbes as a source of nutrients. PlosOne 7, e11915.

Pliego, C., de, Weert, S., Lamers, G., de, Vicente, A., Bloemberg, G., Cazorla, F. M., and Ramos, C. (2008). Two similar enhanced root-colonizing *Pseudomonas* strains differ largely in their colonization strategies of avocado roots and *Rosellinia necatrix* hyphae. *Environ. Microbiol.* 10, 3295–3304.

Ryan, R. P., Monchy, S., Cardinale, M., Taghavi, S., Crossman, L., Avison, L. B., Berg, G., van, der, Leliec, D, and Dow, J. M. (2009). The versatility and adaptation of bacteria from the genus *Stenotrophomonas*. *Nature Rev. Microbiol.* 7, 514−525.

Schmid, M., Iversen, C., Gontia, I., Stephan, R., Hofmann, A., Hartmann, A., Jha, B., Eberl, L., Riedel, K., and Lehner, A. (2009). Evidence for a plant-associated natural habitat for *Cronobacter* spp. *Res. Microbiol.* 160, 608–614.

Tharek, M., Dzulaikha, K., Salwani, S., Amir, H. G., and Najimudin, N. (2011). Ascending endophytic migration of locally isolated diazotroph, *Enterobacter* sp. strain usml2 in rice. *Biotechnology* 10, 521–527.

Verginer, M., Siegmund, B., Cardinale, M., Müller, H., Choi, Y., Miguez, C., Leitner, E., and Berg, G. (2010). Monitoring the plant epiphyte *Methylobacterium extorquens* DSM 21961 by real time PCR and its influence on strawberry flavor*.* *FEMS Microbiol. Ecol.* 74, 136–145.

Wu, C. H., Hwang, Y. C., Lee, W., Mulchandani, A., Wood, T. K., Yates, M. V., and Chen, W. (2008). Detection of recombinant *Pseudomonas putida* in the wheat rhizosphere by fluorescence *in situ* hybridization targeting mRNA and rRNA. *Appl. Microbiol. Biotechnol.* 79, 511–518.
